# Correction: Testing the potential of streamflow data to predict spring migration of ungulate herds

**DOI:** 10.1371/journal.pone.0264402

**Published:** 2022-02-17

**Authors:** Jason S. Alexander, Marissa L Murr, Cheryl A. Eddy-Miller

There is a typographical error in the article title. The correct title is: Testing the potential of streamflow data to predict spring migration of ungulate herds. The publisher apologizes for the error.
