# Supplementary material for: Testing the potential of streamflow data to predict spring migration of an ungulate herds
Source: PLoS One. 2022 Jan 21;17(1):e0262078. doi: 10.1371/journal.pone.0262078 (PMC8782492; doi:10.1371/journal.pone.0262078)
Supplement: Attachment — Submitted filename: Response to Reviewers.pdf [file pone.0262078.s002.pdf]

PONE-D-20-40787
Testing the potential of streamflow data to predict spring migration of an ungulate
PLOS ONE

Dear Editors,

My co-authors and I would like to thank the editors at PLOS ONE and the peer reviewers for taking the time to improve our manuscript. Below you will find our responses organized into three sections, responses to PLOS ONE Academic Editors, responses to Reviewer #1, and responses to Reviewer #2. We have done our best to incorporate the suggested changes but, as you will see in our responses, in some cases the reviewers simply did not provide specific criticisms or detailed comments that would direct us to their concerns. If, after reading our rebuttal, the Academic Editors or Reviewers feel additional work is needed to improve the manuscript, we stand ready to address their revised comments. Our rebuttals to each suggestion or reviewer comment are in red to make them easily identifiable. Please also note that we have included a revised Figure 2. The revision corrected an error in the annotations in the top frame (2A). We also made sure to use PLOS ONE's online image checking tool, and export the figure as at least 300 dpi .tiff file to address reviewer #2's comments regarding its poor quality, which we interpreted as low-resolution. We look forward to working the editorial staff to move this manuscript forward.

Sincerely,

Jason S. Alexander, PhD, Hydrologist, U.S. Geological Survey, Wyoming-Montana Water Science Center

---

Rebuttal to Academic Editors:

When submitting your revision, we need you to address these additional requirements.

1. Please ensure that your manuscript meets PLOS ONE's style requirements, including those for file naming. The PLOS ONE style templates can be found at

https://journals.plos.org/plosone/s/file?id=wjVg/PLOSOne_formatting_sample_main_body.pdf and

https://journals.plos.org/plosone/s/file?id=ba62/PLOSOne_formatting_sample_title_authors_affiliations.pdf

[Author Response]: We have reviewed the style requirements, and believe our manuscript meets the style requirements. We used the PLOS ONE LaTeX template in Overleaf to prepare our manuscript. If there are specific style concerns identified by the editors, we would be happy to address them.

2. Please ensure that you refer to Figure 8 in your text as, if accepted, production will need this reference to link the reader to the figure.

[Author Response]: Good catch! We have inserted a reference to Figure 8 in its proper location (last paragraph of results).

3.We note that Figure(s) 1 in your submission contain map images which may be copyrighted. All PLOS content is published under the Creative Commons Attribution License (CC BY 4.0), which means that the manuscript, images, and Supporting Information files will be freely available online, and any third party is permitted to access, download, copy, distribute, and use these materials in any way, even commercially, with proper attribution. For these reasons, we cannot publish previously copyrighted maps or satellite images created using proprietary data, such as Google software (Google Maps, Street View, and Earth). For more information, see our copyright guidelines: http://journals.plos.org/plosone/s/licenses-and-copyright.

[Author Response]: All of the layers used to create Figure 1 are publicly available at no cost in online databases. We have inserted the following statement in the caption for Figure 1 to make the references clear (Note "[19]" is reference to Kauffman et al, 2020):

"Topographic, hydrologic, and point feature data used to create the map are all freely available from U.S. Geological Survey (https://www.usgs.gov/products/maps/gis-data) and U.S. Department of Agriculture Natural Resources Conservation Service (https://datagateway.nrcs.usda.gov/). Migration paths are from [19]."

Rebuttal to Reviewer #1 Comments:

Reviewer #1: The authors provided an approach to predict the timing of spring migration of mule deer herds and individuals based on the Colorado River's streamflow data. The subject of the paper is interesting, and it has the potential to publish in the PONE. Since the method's application is the only contribution of the current study, in this stage of review, I only focus on the method section. If the paper has a chance for further consideration based on the editor's decision, I will provide other comments after checking the other sections. However, the authors should address several concerns in this stage of review. So, I recommend the paper should be revised as major revision.
- The authors should provide a flow chart to demonstrate the method proposed in the current study step by step.

[Author Response]: We agree that flow charts can be helpful as a visual description of the modeling process and can add clarity for readers. However, please see our responses below regarding the modeling process. We would be happy to build a flow chart as an additional figure but would like to be sure we have addressed the reviewer's concerns with the statistical modeling before doing so.

- The authors used the Kendall-Theil method to assess the trend of the variables employed in the current study. What is the advantage of that method? Is there any other alternative to conduct trend analysis. What is the advantage. I believe the author should use recently developed methods.

[Author Response]: The Kendall-Theil method is a widely accepted non-parametric method to quantify the presence of trends in time-series data. It is insensitive to outliers and does not require an assumption of normally-distributed residuals. It is a method that is widely accepted as accurate and commonly used in hydrologic trend analysis (Helsel et al, 2020). The main concern with the method is autocorrelation in the data. In this case, the data are simple, single-variate time-series of measures of spring phenological development and snowmelt timing. We state in the first paragraph of the 'Modeling' section, that "*The data were checked for autocorrelation before testing for temporal trends, and no significant autocorrelation was detected in any of the series. P-values*". It should be noted that we also tried the typical trend test, simple least-squares linear regression, and it produced nearly identical results. However, that trend test is more sensitive to outliers and can produce biased results, so we chose to use the unbiased model.

If the reviewer has specific concerns with the method (bias, error etc.), we would be happy to address those concerns. Likewise, if the reviewer would like to suggest a method for trend analysis that they believe would provide some different interpretation of trends, we would be happy to try that method.

- The authors used a simple linear regression model for prediction modeling. I believe the relationship between the target and predictive variables is not linear. So, I suggest examining the AI models for prediction.

[Author Response]: We thank the reviewer for their concerns, however, their comments do not provide any technical reasoning supporting their suggestion that our modeling approach is inadequate, nor do they specify an alternative modeling approach that would be an improvement, other than a general suggestion to use AI methods. There are numerous AI methods, many of which are <u>not</u> applicable here. In the 'Discussion' section we suggest that, with additional migration datasets covering a wider range of the landscape, higher level machine learning approaches may be able to extract more information, but such modeling was beyond the scope of this paper. Because the reviewer chose to focus on the methods, it is possible they did not see this discussion.

There are several reasons why we used our modeling approach (logistic regression). They are as follows:

- We adhere to the 'principal of parsimony' in statistical modeling. That is, the model with the fewest explanatory variables and highest explanatory power is the optimum model. This principal is rooted in the philosophy that statistical modeling is strengthened by making inference on model parameter estimates, which inevitably becomes more difficult in higher-order models because of the effects of multi-collinearity in predictor variables.
- Our dataset essentially has 10 observations from each herd (migrating =1 or not =0), which means that the ratio of explanatory variables to observations is a minimum of 1:10 (for each herd), and as high as 3:10. So adding additional co-variates is not appropriate here because this would cause those models to be overparameterized.
- As explained in the first paragraph of the 'Materials and Methods' section, we chose logistic regression as our modeling approach because we wanted to represent migration as a probability. Through exponentiation of the odds ratio, logistic regression has a clear and direct mathematical translation to probability. AI methods do not have this straightforward relationship with probability.
- More 'modern' AI (machine learning) methods are designed to extract information from models which have very large numbers of explanatory variables, typically >>3. Of the 20 models presented in our analysis, 12 have 1 explanatory variable, 4 have 2 explanatory variables, and 4 have 3 explanatory variables. As described in the text, and shown in table 1, the multi-variate models do not have remarkably greater accuracy or explanatory power than the single-variate models.
- Although logistic models are fully capable of describing non-linear processes, the processes we are trying to model here are remarkably linear. Below is a figure we prepared using simple, least-square linear regression (regression on a continuous variable), which demonstrates that timing of onset of the annual spring pulse is linearly related to the timing of onset of mule-deer migration. We did not use this figure because we chose to use logistic regression instead of least-squares linear regression for the reasons stated above.

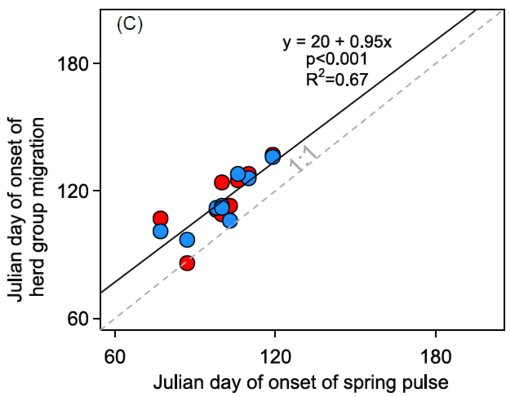

Our modeling results (summarized in Table 1, and figures 6,7, and 8) demonstrate that our modeling approach adequately represents the process, with little error (RMSE of +/- 7 days). If the reviewer would like to provide specific comments that point to evidence of bias or error in our modeling approach, evidence that our modeling efforts are not representing the process (timing of spring migration in individuals and groups of deer in two different herd), or want to suggest an alternative method that could more accurately model the process, while still adhering to the principal of parsimony, we would be happy to address those concerns.

It should be noted that, although not the newest vintage, logistic regression is a so-called "AI" method. It can also deal with and is widely used to model non-linear processes such as landslides and avalanches. So, even though the process here is clearly linear, there is no reason why logistic regression could not be used to model non-linear processes.

- Please update the results base on the mentioned comments as well.

[Author Response]: Please see our responses above.

Rebuttal to Reviewer #2 Comments:

Reviewer #2: In the present paper, the authors applied the Logistic-regression model for modelling the timing of spring migration of mule deer herds. The goal fixed by the authors was clear and objectively justified: predicting the onset of spring migration for herds of mule deer in south-central Wyoming. One of the most significant results of the present investigation is that the streamflows data was more suitable as predictor compared to the temperature data for predicting past and future migrations. The paper is well written, scientifically sound and the originality is evident as the research gap is clearly presented and argued. Clear explanation of methods, results and discussion, and the topic of the manuscript are very important. As such, this manuscript definitely deserves to be published

in the PLOS ONE Journal. I have only one fewer suggestions to the authors:

1. Page 5/14 Lines: For the purposes of modeling we defined the day of herd migration initiation as the first day the maximum number of deer in each herd were in spring migration. I think this sentence needs to be reformulated.

[Author Response]: We agree that this sentence may be confusing. We have re-worded this sentence to read

"We arbitrarily defined the day that groups of deer (herds) initiated migration as the first day of each year j the maximum number of collared deer in each herd were migrating simultaneously."

In addition, the sentence that follows the one above should also help clarify the definition

"That is, if the maximum number of deer migrating simultaneously in the northern herd in year j was 7, then MH_{ji} was set to 1 on the first Julian day 7 deer in the northern herd were simultaneously in spring migration."

We hope that the reviewer finds more clarity in these definitions.

2. Figures 3 and 7 (Scatterplot) provides the R2 and the regression equation.

[Author Response]: Figures 3 and 7 show 'predicted versus observed' relationships and thus are not models. Thus, showing equations (models) and coefficient(s) of determination would not be appropriate in these figures.

3. Figure 2 should be improved it is for bad quality and unclear.

[Author Response]: The reviewer's comment of 'bad quality' and 'unclear' do not provide us with any specific concerns to address in figure 2. We interpret these comments to mean that the resolution of the figure received by the reviewer was of low quality (i.e. not >=300 dpi)? We provided PLOS ONE with a high quality (>=300 dpi) figure. So, if the reviewer's concerns are with the resolution of the figure, that is an easy fix (please provide them with the figure we provided PLOS ONE). If there are other specific concerns (color scheme is confusing or annotations are unclear/incorrect), we would be happy to address them, but we currently do not have enough information from the reviewer to make changes to improve figure 2.

References Cited

Helsel, D.R., Hirsch, R.M., Ryberg, K.R., Archfield, S.A., and Gilroy, E.J., 2020, Statistical methods in water resources: U.S. Geological Survey Techniques and Methods, book 4, chapter A3, 458 p., https://doi.org/10.3133/tm4a3
