# Supplementary material for: Testing the potential of streamflow data to predict spring migration of an ungulate herds
Source: PLoS One. 2022 Jan 21;17(1):e0262078. doi: 10.1371/journal.pone.0262078 (PMC8782492; doi:10.1371/journal.pone.0262078)
Supplement: Attachment — Submitted filename: Response to Reviewers.docx [file pone.0262078.s004.docx]

PONE-D-20-40787R2
Testing the potential of streamflow data to predict spring migration of an ungulate
PLOS ONE

Dear Editors,

My co-authors and I are happy to re-submit our manuscript ‘Testing the potential of streamflow data to predict spring migration of an ungulate’ to PLOS ONE. We thank the editors for identifying high-quality reviewers whose expertise lies in movement ecology, which is a large focus of the manuscript. We found the most recent review comments were very helpful, and greatly improved the quality and clarity of the manuscript. Please find our responses to reviewers below in red font, each identified with the title ‘[Author response]’. Changes to the manuscript can be found in the document “Revised Manuscript with Track Changes.pdf”, where red strikethrough text indicates removal, and blue text indicates insertion. Because the manuscript was written in Overleaf in the LaTex language, we used the program ‘LaTexDiff’ to track changes. This program is the only option for tracking changes in these types of files, and it sometimes has problems. Table 2, for example, does not fit on the page when changes are noted, and so you will not be able to see all of the changes there. However, the only changes to that table were the order of the models, NONE of the contents of the table changed.

As part of the U.S. Geological Survey’s publishing policy, we were also required to respond to a review from a qualified USGS reviewer. That reviewer was Dr. Tabitha Graves, whose expertise lies in landscape ecology and wildlife biology. You can find out more about Dr. Graves at her [staff website](https://www.usgs.gov/staff-profiles/tabitha-graves?qt-staff_profile_science_products=0#qt-staff_profile_science_products). I have taken the liberty of pasting her review letter, and our responses to her comments below the PLOS one reviewer comments. As you will see, Dr. Graves comments were mainly associated with clarifying some technical terms, and she had no major issues with the methods, results, or conclusions. So her comments did not result in any major changes to methods, results, or interpretations, but changes were made to some of the terminology in the text, and some redundant text was removed. Likewise, slight changes were made in terminology after a review by our government approving official. All of those changes were all editorial and will not affect interpretations. Minor editorial changes were also made to figures 1,2,3, and 4 based on comments from our approving official. Again, all of these changes were editorial and made to annotations or, in the case of figure 1, additional of coordinate ticks at the edge of the map.

We hope you find the revised manuscript greatly improved, and suitable for acceptance for publication in PLOS ONE.

Best,

Jason S. Alexander PhD

---------------REVIEW COMMENTS AND RESPONSES BELOW-------------------------------

**Comments to the Author**

1. If the authors have adequately addressed your comments raised in a previous round of review and you feel that this manuscript is now acceptable for publication, you may indicate that here to bypass the “Comments to the Author” section, enter your conflict of interest statement in the “Confidential to Editor” section, and submit your "Accept" recommendation.

Reviewer #3: (No Response)

Reviewer #4: (No Response)

 2. Is the manuscript technically sound, and do the data support the conclusions?

The manuscript must describe a technically sound piece of scientific research with data that supports the conclusions. Experiments must have been conducted rigorously, with appropriate controls, replication, and sample sizes. The conclusions must be drawn appropriately based on the data presented.

 Reviewer #3: Partly

Reviewer #4: Yes

 3. Has the statistical analysis been performed appropriately and rigorously?

Reviewer #3: I Don't Know

Reviewer #4: Yes

 4. Have the authors made all data underlying the findings in their manuscript fully available?

The [PLOS Data policy](https://gcc02.safelinks.protection.outlook.com/?url=http%3A%2F%2Fwww.plosone.org%2Fstatic%2Fpolicies.action%23sharing&data=04%7C01%7Cjalexand%40usgs.gov%7C58c1bfabeb204b4b0bbf08d93fc3cba3%7C0693b5ba4b184d7b9341f32f400a5494%7C0%7C0%7C637610932448156862%7CUnknown%7CTWFpbGZsb3d8eyJWIjoiMC4wLjAwMDAiLCJQIjoiV2luMzIiLCJBTiI6Ik1haWwiLCJXVCI6Mn0%3D%7C1000&sdata=hX7vLfVseCab9vuaP52dh2UrYCBFDM7XRJgT9NQa9fY%3D&reserved=0) requires authors to make all data underlying the findings described in their manuscript fully available without restriction, with rare exception (please refer to the Data Availability Statement in the manuscript PDF file). The data should be provided as part of the manuscript or its supporting information, or deposited to a public repository. For example, in addition to summary statistics, the data points behind means, medians and variance measures should be available. If there are restrictions on publicly sharing data—e.g. participant privacy or use of data from a third party—those must be specified.

 Reviewer #3: Yes

Reviewer #4: Yes

 5. Is the manuscript presented in an intelligible fashion and written in standard English?

PLOS ONE does not copyedit accepted manuscripts, so the language in submitted articles must be clear, correct, and unambiguous. Any typographical or grammatical errors should be corrected at revision, so please note any specific errors here.

Reviewer #3: Yes

Reviewer #4: Yes

 6. Review Comments to the Author
----------------------REVIEWER #3 COMMENTS AND AUTHOR RESPONSES----------------
**Reviewer #3:** The authors provided a new method to predict female mule deer migration onset from streamflow data which are available in real time and for long term time series, undoubtedly opening interesting perspectives for wildlife management. The idea is very original and the logical flow easy to follow. Nevertheless, in my opinion some points should be clarified before publication. The reported anticipating trend of migration onset should be statistically justified, but my impression is that the potential to predict future (rather than past) migrations is the most important and useful finding of the manuscript. Below you can find more specific comments on major and minor issues to be addressed.

Major issues:
[REVIEWER #3 COMMENT] Introduction and Discussion chapters miss information regarding cues of ungulate migrations. The reported past and future predictions on mule deer migration onset is justified only if environmental cues (temperature, snowmelt, vegetation growth…all variables that authors cleverly summarized within the streamflow) are the only (or main) cue triggering migration onset. Nevertheless, phenology of northern latitudes ungulate species typically (mostly) relies on photoperiodism, which is independent form environmental changes. This is the reason for global change-driven mismatches between migration and optimal foraging conditions provided within the green wave (e.g., Stenseth and Mysterud 2002; Mysterud 2013) and the consequential loss of fitness in migrating ungulates (mentioned by the authors in L46). Notwithstanding, there are evidence of high environmental plasticity of the migration timing, for example in caribou which mostly trigger migration with snowmelt (Laforge et al. 2021). The results of this manuscript potentially provide a similar evidence, but this issue should be introduced and discussed within its broader chronobiological context.
[AUTHOR RESPONSE]:

- The reviewer is correct that photoperiodism plays a role in migration of many species. However, if photoperiodism were a primary driver of ungulate migrations in high latitude or high-altitude areas, there would not be such plasticity in migration timing observed in many ungulate species because the timing of changes in daylight length at any latitude is essentially constant over geologically short periods. There is abundant evidence that many migratory ungulates in northern latitudes exploit resource waves (the so-called ‘green wave’) along latitudinal and elevational gradients to maximize nutritional uptake in the spring. Merkle et al. (2016), for example, showed that 7 of 10 species they examined were surfers of the green wave.
- In mule deer of Wyoming, there is additional evidence that, although they could complete their migration in a matter of days, these animals spend weeks at stopover feeding grounds along their routes, tracking the highest nutritional content of the resource (Sawyer and Kauffman, 2011). There is also evidence that, even during droughts when spring is shorter and the green wave moves faster, mule deer can track the most nutritionally rich edge of the resource wave (Aikens et al., 2020).
- We recognize that the reviewer’s comment is likely a reflection of our poor framing of the problem in the Introduction, and a lack of detail in the discussion. We have put the following language into the *Introduction* and *Discussion* in the hopes that it improves the set-up of the problem and enriches the discussion. New language is highlighted in yellow, and location is identified by section and paragraph:
  - [INTRODUCTION, first paragraph]: “*Exploitation of resource waves has been widely documented as a crucial component in the life history of migratory herbivores [1]. In northern latitudes, so-called ‘green-wave’ migrations are caused by spatially-progressing greening of the landscape along elevation and latitude gradients associated with plant phenological development in the transition from spring to summer [2-5]}. The green wave hypothesis is a subset of the broader forage-maturation hypothesis which states that ungulates trade off forage quantity for forage quality to maximize energy intake [6]. In mountainous and high latitude regions of North America, the onset of the green wave coincides with rising daily mean temperatures causing snowmelt and subsequent progressive exposure of soil and vegetation along elevation gradients.”*
  - [INTRODUCTION, second paragraph]: “*The satellite-derived normalized-vegetation difference index (NDVI) and associated derivatives, for example have been used to model spatial gradients in plant crude protein, making inference on benefits of surfing strategies such as trailing, surfing, or jumping the green wave [3,9-11]. Bischof et al (2012) used satellite-derived NDVI to observe that male and female red deer (Cervus elaphus) moved rapidly from summer to winter grounds, jumping the green wave. Similarly, LaForge et al. (2021) [12] used time-series of the instantaneous rate of green up (IRG), an NDVI-derived metric, and observed that female caribou (Rangifer trandus) jumped the greenwave by 1 week, and maximum greenup in summer feeding grounds occurred after calving. Alternatively, Merkle et al. (2016) [2] examined surfing behaviors of a host of ungulates in mountain regions of the western United States and found that, despite some variation, a majority of species migration paths tracked high IRG areas (i.e. surfed the green wave).”*
  - [INTRODUCTION, third paragraph]: “*Satellite-derived data such as NDVI, IRG, and snowcover, however, have a limited period-of-record and can have high-latency at the daily time step due atmospheric interference (i.e. MODIS). While typically less limited in period-of-record, temperature-derived measures…”*
  - [DISCUSSION, NEW third paragraph]: *““There are inherent assumptions in our models which add complexity to interpretation of our results. The modeled prediction of a median shift in migration initiation of 8 days earlier than the mid-20th century for groups of female mule deer is just beyond the calculated error of the best model (RMSE = 7.4 days). That model relied only on the timing of snowmelt initiation, and thus was strongly influenced by the underlying, but significant, temporal trends of that data. Modeling temporal trends in the timing of female mule deer migration over the past 85 years required assumptions that these herds have been responding to the same environmental cues over that period, that no major shifts in migratory behaviors have occurred (i.e. switch between resident and migratory), and that locations and distances between each herd's summer and winter feeding grounds has not changed. Given that land uses, land and herd management, and vegetation across this landscape has undoubtedly changed over that time period, this assumption is potentially naive. Research by Sawyer et al. (2019) [37] has shown that some ungulates, particularly mule deer in the mountainous western United states, have little plasticity in migratory routes, indicating some of our assumptions are reasonable for these species, but may not hold for species with higher migratory route plasticity. Recent modeling by Barnhardt et al. (2020) [38] has indicated that snowmelt timing and rate are modulated by plant-available water and available energy, which adds complexity to the assumption that the response of spring plant phenology to snowmelt has remained constant over the modeled historical period. Nonetheless, the extensive and continuous periods of record at some streamgages invites potential for investigating temporal changes in migration initiation that may assist in understanding population dynamics of migrating ungulates which exploit the green wave.”*

- The onset of spring migration by individuals (MI) is biased by the variability in the number of individuals monitored for each year and herd. With the described method, larger samples will inevitably have a higher likelihood to pick smaller minimum values (in this case, earlier dates). MIji is in other words not comparable among years and herds (and indeed seems anticipated in SAR, the herd with more monitored individuals). As individual and herd migration dates are inconsistent (i.e., when comparing SAR and NAR) and predictions have been rightly based only on herd migration (MH) I would suggest removing any mention of MI from the manuscript.

[AUTHOR RESPONSE]:

- The reviewer is correct in their assertion that there is potential for bias in migration date between individuals in each herd based on sample size.
- However, we would assert that the smaller sample size is more likely to affect the precision of the estimate, rather than the accuracy.
- If the estimates of the earliest migration day were affected by sample size, then that would also be true of estimates *within* each herd. So, to help alleviate the concerns of bias by the reviewer, we bootstrapped estimates of the mean-minimum (earliest) migration day for the southern herd by sampling migration initiation days (with replacement, 100 iterations) using the same sample size of the northern herd for each year. The results of the experiment are shown in the plot below.

- Two patterns emerge in the plot above:

(1) Within each herd, sample size appears to have only a weak effect on the estimate of the earliest migration day (i.e. larger samples within each herd do not necessarily mean earlier migration dates.). For example, there is a range of nearly 30 days for samples sizes ~15 in the northern herd, and a range of nearly 50 days for samples sizes ~20 in the southern herd. For very small sample sizes (n=1), there is some leverage on the migration day, but overall, the pattern is weak.
(2) The blue hollow circles corresponding to the sample size of the red dots show the bootstrapped estimate of the mean-earliest migration day (southern herd) for the same year and same sample size as the red dots (northern herd). In 7 out of 8 cases, the bootstrapped means of the southern herd (hollow blue circles) are earlier than the corresponding means of the same sample size for the northern herd (red dots). The mean of the bootstrapped earliest day across all years was, in fact, later than the mean of the observed, but only by 6 days, which is within the RMSE of the logistic model reported in the text, and thus within the precision of the method. Likewise, the mean of the maximum earliest bootstrapped estimates (shown as the upper edge of the purple polygon in the plot) does not overlap with the mean of observed values for the northern herd, again indicating the observed earlier migration times of the southern herd are real.
(3) We recognize that the concerns of the reviewer are also likely to arise with the journal audience. To address concerns of bias due to sample size we have inserted the following statement into the discussion. Changes to the text are highlighted in yellow:
    a. [DISCUSSION, first paragraph] “The models included in the analysis were, intentionally, simple, and were constructed mainly to compare performance of models using streamflow records with those using more traditional measures of spring to predict past and future green-wave migration timing of female mule deer. Given the limited data available data, the models appear to have also accurately reproduced observed migration behaviors among groups and individuals within the NAR and SAR herds, with the best models predicting individuals in the SAR herd have higher probability of initiating migration earlier in the year. It is possible that the larger sample size of the SAR herd in most years biased those data toward earlier migration dates. However, a bootstrap analysis using the sample size of the northern herd indicated sample size had little effect on the mean earliest migration initiation timing in the SAR relative to the NAR.”

- Predictions of past migrations (i.e., occurred between 1943 and 2005) assume that the importance of environmental cues (in respect to photoperiodism, see previous comment) in triggering mule deer migrations has remained unchanged since 1943 despite the ongoing climate change. This can be a reasonable assumption but, in my opinion, should be explicitly communicated to readers, as we cannot exclude that mule deer population became more and more capable of using environmental cues (and not photoperiodism) to trigger migration.

[AUTHOR RESPONSE]:

- This is an excellent point and as suggested we have inserted the following text into the manuscript to address this issue. Note that this text was also presented above to address the reviewer’s comment regarding the need for more context in the Introduction and Discussion sections. Again, new text is highlighted yellow:
  - [DISCUSSION, NEW third paragraph]: *“Although our model parameter estimates are significant and accurately reproduce migration behaviors in the NAR and SAR herds, our sample size was relatively small (n=17), and there are inherent assumptions in our models which add complexity to interpretation of our results. The modeled prediction of a median shift in migration initiation of 8 days earlier than the mid-20th century for groups of female mule deer is just beyond the calculated error of the best model (RMSE = 7.4 days). That model relied only on the timing of snowmelt initiation, and thus was strongly influenced by the underlying, but significant, temporal trends of that data. Modeling temporal trends in the timing of female mule deer migration over the past 85 years required assumptions that these herds have been responding to the same environmental cues over that period, that no major shifts in migratory behaviors have occurred (i.e. switch between resident and migratory), and that locations and distances between each herd's summer and winter feeding grounds has not changed. Given that land uses, land and herd management, and vegetation across this landscape has undoubtedly changed over that time period, this assumption is potentially naive. Research by Sawyer et al. (2019) [37] has shown that some ungulates, particularly mule deer in the mountainous western United states, have little plasticity in migratory routes, indicating some of our assumptions are reasonable for these species, but may not hold for species with higher migratory route plasticity. Recent modeling by Barnhardt et al. (2020) [38] has indicated that snowmelt timing and rate are modulated by plant-available water and available energy, which adds complexity to the assumption that the response of spring plant phenology to snowmelt has remained constant over the modeled historical period. Nonetheless, the extensive and continuous periods of record at some streamgages invites potential for investigating temporal changes in migration initiation that may assist in understanding population dynamics of migrating ungulates which exploit the green wave.”*

- The anticipating trend of migration onset from 1943 to 2018 should be statistically supported rather then only described in terms of medians of arbitrary periods of 20 years. As a matter of example, just suppose that this study was conducted in 1979: authors would have concluded that along 36 years, from 1943 to 1979, the migration onset had been delayed of 5 days (rather than anticipated because of climate change). Looking at Figure 8, my impression is that the variability of predicted migration onset dates among years, together with their uncertainty, do not support the conclusion of a tendency to anticipate migrations over years (but my impression could be proven wrong with a proper statistical test).

[AUTHOR RESPONSE]:

- The reviewer is correct that, in time series, the choice of beginning time period can affect the trend detected. However, the trend of the independent explanatory variable (day of snowmelt onset) is statistically significant (see Figure 4c), and the diagnostics on the model used to generate the prediction of migration onset are statistically significant (Bayesian probability interval for parameter estimate does not include 0). Thus we would suggest the predictions would also be expected to be significant.
- We would suggest that running a classical statistical hypothesis test (which we think is what the reviewer is asking) on the results of a model that was informed by a variable with a significant temporal trend, and which is the model with the smallest errors (see table 2) would be redundant (significance is known).
- A better measure of error is the RMSE of the model residuals, which is 7.4 days, whereas the median increase over the time period is just outside of these bounds (8 days), and the largest increase is as much as 13 days. So, despite being a small difference, the model already demonstrates significance.
- We realize that the lack of clarity regarding errors versus predictions expressed by the reviewer’s comment may also be experienced by the audience of PLOS ONE. To alleviate this concern, we have inserted the following language into the discussion Note that some of this text was also presented above to address the reviewer’s comment regarding the need for more context in the Introduction and Discussion sections. Again, new text is highlighted yellow:

- [DISCUSSION, NEW part of third paragraph]: *Although our model parameter estimates are significant and accurately reproduce migration behaviors in the NAR and SAR herds, our sample size was relatively small (n=17), and there are inherent assumptions in our models which add complexity to interpretation of our results. The modeled prediction of a median shift in migration initiation of 8 days earlier than the mid-20th century for groups of female mule deer is just beyond the calculated error of the best model (RMSE = 7.4 days). That model relied only on the timing of snowmelt initiation, and thus was strongly influenced by the underlying, but significant, temporal trends of that data.*

Minor issues:
- Introduction miss information about the known migration patterns of the monitored species. In particular:
• Do mule deer behave as a green wave “surfer” or “jumper”? This information is needed to understand if they can simply follow the green wave or rather must anticipate it with reliable cues.

[AUTHOR RESPONSE]: Thank you to the reviewer for pointing out that additional description of the green wave surfing dynamics is needed so the reader has a complete context for the migration ecology of the mule deer populations used in this study. We have added the following text to the ‘Study Area’ section, where we believe a description of the specific behaviors of the NAR and SAR herds is most appropriate. New text is highlighted yellow:

- [Study Area, first paragraph]: “In keeping with previous studies [24-26], these populations are respectively referred to as the northern and southern Atlantic Rim herds (*herein* NAR and SAR herds, respectively). Female mule deer in these herds have been studied extensively and have been shown to be surfers of the green wave, have very few residents, and low plasticity in their migration routes [2,25,27]. “

• Does the whole population migrate every year, or a proportion of individuals are stationary? Is this eventual proportion constant among years or rather it varies depending on environmental conditions? This is important to understand the biological meaning of the estimated MH.

[AUTHOR RESPONSE]: Please see answer to previous comment.

• Do females and males migrate together? Or, in other words, are female migration patterns a good proxy of the whole population migration? As only female mule deer has been monitored, this should be stated and has major management implications. If there are no reasons to consider female migration as a reliable proxy of the whole populations, the authors should specify throughout the text that their results only refer to female mule deer.

[AUTHOR RESPONSE]: There is little, if any, data on male migration in these populations, just anecdotes from studies of these herds. We have added the following text to the ‘Study Area’ section, where we believe a description of the specific behaviors of the NAR and SAR herds is most appropriate. New text is highlighted yellow:

- [Study Area, first paragraph]: “Although female deer in these herds have been the primary focus of study, past research has noted that spatial segregation between male and female deer occurs at a fine scale, and that migration patterns among males are likely similar to females [28]. Nonetheless, the analysis, results, and conclusions presented in this paper apply only to female mule deer because no male mule deer were tracked.”

- Just for clarity, how was the database organized for the logistic regression models? If I understood correctly, it was organized with 151 records per year (one for each considered Julian date) assuming 0 from 1st January until the MH date of the reference year and 1 from MH date onward. Please consider rephrase the corresponding Methods section to clarify.

[AUTHOR RESPONSE]: This is an excellent point we have added the following text to those lines in the methods to clarify database organization (new text highlighted in yellow):

*“We applied these rules to the first 151 days of the year whereby all days prior to migration initiation were set to a value of 0, and all days after initiation up to Julian day 150 were set to 1. This 151 day period, although arbitrary, was chosen because it is the end of May in common years, and encompassed the latest calculated $GDD_{180}$ and $GDD_{jerk}$ (147), latest day of spring pulse initiation (119), and latest day of initiation of group migration within mule deer herds (137)”*

- Figure 6: y axes are named (slightly) different in A and B. In addition, it is unclear why predictions were made for the period 2003-2018, while observations counted only 9 years and started in 2005. Please clarify.

[AUTHOR RESPONSE]:

- Thank you for pointing out the error in the y-axes. We have corrected y-axis of the upper plot to match the y-axis of the lower plot.
- The caption stating ‘2003 to 2018’ is a typo. We used the period of record from 2002 to 2018 to generate the probability bounds. We have made that correction to the caption, and the one location where it is mentioned in the text. We have also inserted a statement in the caption to help clarify (new text is highlighted):

*“The period of record used to generate the probabilities was 2002 to 2018, which is the most recent continuous period of record. Water years 2000 and 2001 had incomplete records.* *The observations of initiation of spring migration for individuals and herds of mule deer are from years 2005-2006, 2008-2010, 2015-2018.”*

- To clarify our choice of period of record in the text, we modified the existing statement to read as follows (new text is highlighted):

*“Model 5 (Table 2) was used to generate probabilities of initiation of spring migration for mule deer herds and individuals in the NAR and SAR herds for calendar years 2002 to 2018. This period of record was chosen because it is the most recent continuous period of record of daily streamflow at the Slater gage overlapping with the period of mule deer migration observations”*

- To be clear, we used the observed data to train the model (model 5) and obtain parameter estimates, then used the model to predict probability bounds, and compared those to the observed data. This allowed some validation check between the observed data used to train the model, and the probability bounds for the entire 2002 to 2018 POR.

  Literature cited:
  Laforge, M. P., Bonar, M., & Vander Wal, E. (2021). Tracking snowmelt to jump the green wave: phenological drivers of migration in a northern ungulate. Ecology, 102(3), e03268.

  Mysterud, A. (2013). Ungulate migration, plant phenology, and large carnivores: The times they are a‐changin'. Ecology, 94(6), 1257-1261.

  Stenseth, N. C., Mysterud, A., Ottersen, G., Hurrell, J. W., Chan, K. S., & Lima, M. (2002). Ecological effects of climate fluctuations. Science, 297(5585), 1292-1296.

----------------------REVIEWER #4 COMMENTS AND AUTHOR RESPONSES----------------
Reviewer #4: In this ms, Alexander and colleagues test the applicability of hydrological measurements informed by streamflow data to assess their potential in predicting the timing of migration of mule deer in North America. I received a revises version of the ms, for further revision. I liked the ms, which combines two field of research (movement ecology and hydrology) in a promising way. I think it deserves publication in PlosONE. It is well written, it clearly states the problem, it has a strong logical structure, and it presents interesting results. As movement ecologist, I don’t feel competent to judge the technical details of the methodological process concerning the hydrological data. Instead, I have very minor suggestions about the ‘ecological side’ of the work, that I hope can further improve the quality of the ms.

1) First line of the abstract: I would remove ‘of North America’: green wave surfing is a movement tactic that applies to ungulates worldwide

[AUTHOR RESPONSE]: We agree that these migrations occur worldwide and many latitudes. We have taken the reviewers suggestion and removed ‘North America’ from the sentence.

2) LL 18-20: when you speak about NDVI applied to measure surfing capacity by ungulates, I would recommend to cite the following work:
Aikens, E. O., Kauffman, M. J., Merkle, J. A., Dwinnell, S. P., Fralick, G. L., & Monteith, K. L. (2017). The greenscape shapes surfing of resource waves in a large migratory herbivore. Ecology Letters, 20(6), 741-750.

[AUTHOR RESPONSE]: Agreed, we have inserted the reference.

3) LL 69-71: “Logistic regression models were used to examine the potential to predict initiation of spring migration of individuals and groups of mule deer in the same broader river basin”. Please replace ‘groups’ by ‘herds’

[AUTHOR RESPONSE]: We understand there can be some confusion between the term ‘group’ and ‘herd’. However, as the reviewer can probably infer from the manuscript, we are hesitant to assign the word ‘herd’ to the groups of individuals which were observed migrating all at once. This is because we do not want to confuse the reader or assume that the groups of radio-collared deer within the each of the larger herds are representing the entire herd. That may, in fact, be the case, but we don’t know for sure, so we would prefer to keep using the term ‘group’. We will change it if the reviewer feels it is imperative, but will leave that decision to the editor.

4) LL 164-165: “Logistic regression models were used to assess the relative potential of explanatory variables to predict onset of spring migration of individuals (MIi) and groups (MHi)”. I think that it would be appropriate to move here in this section the issue that you raise about correlation between covariates (instead of presenting it in the Results, LL 229-230): such way, the reader comes to the results sections with a bit clearer idea about what models have been fitted and compared.

[AUTHOR RESPONSE]: We agree that a statement in the methods regarding testing of multi-collinearity between explanatory variables is warranted. We have inserted the following statement at the end of the second paragraph of the ‘modeling’ section to make the reader aware that we were aware of this potential:

*“Pearson's R was used to examine the potential effect of multicollinearity on model explanatory variables, with R values greater than 0.20 between explanatory variables considered to have high potential for multicollinearity”*

5) Results of the models (lines 226 and following): I would suggest separating the part of results where describe the outcome of models for group migration vs individual migration. It seems that you start with a sort of ‘mix’ (“The models including growing degree day (GDD) and the day of snowmelt initiation for the Slater streamgage (Dslater sm ) had the best overall performance for groups and individuals of mule deer”); then, you move to describe the group migration results, but this is not immediate to catch, and only later it becomes clear (i.e. when you move to the individuals results description). It is just a recommendation to help clarity of presentation.

[AUTHOR RESPONSE]: We agree the sentence in this paragraph referring to modeling results for individuals may introduce some confusion because the first two paragraphs of this section generally discuss results of models for groups of female mule deer. We have taken that sentence out of the first paragraph in the section and put it in the third paragraph, where we address results of models for individual female mule deer.

6) Caption to Figure 5: “Herd - dummy variable identifying northern or southern Atlantic Rim herds” ◊ Although you say it in the main text, I would specify also here what is the reference category
[AUTHOR RESPONSE]: We are not quite sure what the reviewer is referring to here. We think they would like us to insert an explanation of where these data were used (as a dummy variable). We have revised the caption of figure 5 to read the following (highlighted text was added):

*“Scatterplot showing comparison of the Julian day of onset of spring migration for: (A) individuals, and (B) groups of radio-collared female mule deer within the northern (NAR) and southern Atlantic Rim herds (SAR). All data are for the years 2005 to 2006, 2008 to 2010, and 2015 to 2018. The days shown in (A) are the earliest migration day of a radio-collared individual female mule deer within each herd. The days shown in (B) are the earliest day when the maximum number of radio-collared female mule deer were migrating simultaneously. A dummy variable was used in the logistic regression model to test for differences in probability of migration initiation for individuals and groups within the northern and southern Atlantic Rim herds, with NAR=1 and SAR=0.”*

7) Table 2: I suggest to present the list of models based on their ranking for precision, it is easier to follow:

[AUTHOR RESPONSE]: We agree with this suggestion. We have changed the table so that the models are listed in order of highest precision, lowest DIC combination. We have, however, preserved the split in the table for groups and individuals, which we believe is important for clarity. We have also added the following statement to the caption for clarity:

*“Model numbers correspond to the order the models were ran. Models for groups and individuals are presented separately and by priority of highest precision and highest recall, and lowest DIC.”*

8) LL 263-265: “As stated previously, we were most interested in representing spring migration initiation of mule deer as a probability because we believe it is the most reasonable representation of data which are, at best, under-informed.” ◊ I think this sentence is useless and it does not add any information, I would remove it.

[AUTHOR RESPONSE]: We concur and have removed the statement from the text.

9) L301 and following: I appreciate the tentative to discuss the different results obtained for the two streamgates. Do you think this could also due to a simple issue of distance between the streamgate and the tracking animals? If this is the case, you could add a sentence to recommend to use data from streamgages as close as possible to the monitored population; or, the other way round, you could address this issue as a potential limitation of the predictive capability of this approach, vs satellite derived measures which are instead by definition insensitive to this potential issue.

[AUTHOR RESPONSE]: We agree that some discussion of distance of streamgage, and how well a streamgage represents the landscape of the migratory animal is in order. We have added the following text to the discussion (new text is highlighted):

*“It is also possible that the runoff dynamics at* $D_{sm}^{slater}$ *more accurately represented the progression of the green wave along the migration routes and\or near the summer feeding grounds of the Atlantic Rim mule deer herds. Additional work is needed to test relations between migration initiation in other herds with networks of gages in basins along or near migration routes and feeding grounds.”*

7. PLOS authors have the option to publish the peer review history of their article ([what does this mean?](https://gcc02.safelinks.protection.outlook.com/?url=https%3A%2F%2Fjournals.plos.org%2Fplosone%2Fs%2Feditorial-and-peer-review-process%23loc-peer-review-history&data=04%7C01%7Cjalexand%40usgs.gov%7C58c1bfabeb204b4b0bbf08d93fc3cba3%7C0693b5ba4b184d7b9341f32f400a5494%7C0%7C0%7C637610932448166817%7CUnknown%7CTWFpbGZsb3d8eyJWIjoiMC4wLjAwMDAiLCJQIjoiV2luMzIiLCJBTiI6Ik1haWwiLCJXVCI6Mn0%3D%7C1000&sdata=3owVTKLda7%2BFSQAmEu6KQ2xOfHDbIE%2FHBpNZtCd9gM8%3D&reserved=0)). If published, this will include your full peer review and any attached files.

If you choose “no”, your identity will remain anonymous but your review may still be made public.

**Do you want your identity to be public for this peer review?** For information about this choice, including consent withdrawal, please see our [Privacy Policy](https://gcc02.safelinks.protection.outlook.com/?url=https%3A%2F%2Fwww.plos.org%2Fprivacy-policy&data=04%7C01%7Cjalexand%40usgs.gov%7C58c1bfabeb204b4b0bbf08d93fc3cba3%7C0693b5ba4b184d7b9341f32f400a5494%7C0%7C0%7C637610932448166817%7CUnknown%7CTWFpbGZsb3d8eyJWIjoiMC4wLjAwMDAiLCJQIjoiV2luMzIiLCJBTiI6Ik1haWwiLCJXVCI6Mn0%3D%7C1000&sdata=EzE9hKc9cBpUKAoQFSj2MiOMBdDAFEqoYklavZ6rzP0%3D&reserved=0).

 Reviewer #3: **Yes:** Rudy Brogi

Reviewer #4: No

 [NOTE: If reviewer comments were submitted as an attachment file, they will be attached to this email and accessible via the submission site. Please log into your account, locate the manuscript record, and check for the action link "View Attachments". If this link does not appear, there are no attachment files.]

While revising your submission, please upload your figure files to the Preflight Analysis and Conversion Engine (PACE) digital diagnostic tool, [https://pacev2.apexcovantage.com/](https://gcc02.safelinks.protection.outlook.com/?url=https%3A%2F%2Fpacev2.apexcovantage.com%2F&data=04%7C01%7Cjalexand%40usgs.gov%7C58c1bfabeb204b4b0bbf08d93fc3cba3%7C0693b5ba4b184d7b9341f32f400a5494%7C0%7C0%7C637610932448176777%7CUnknown%7CTWFpbGZsb3d8eyJWIjoiMC4wLjAwMDAiLCJQIjoiV2luMzIiLCJBTiI6Ik1haWwiLCJXVCI6Mn0%3D%7C1000&sdata=X6nebpWAJkJ7PBG6MBTPUaM31CTc%2BpZ5AjrD0GDBQYo%3D&reserved=0). PACE helps ensure that figures meet PLOS requirements. To use PACE, you must first register as a user. Registration is free. Then, login and navigate to the UPLOAD tab, where you will find detailed instructions on how to use the tool. If you encounter any issues or have any questions when using PACE, please email PLOS at [figures@plos.org](mailto:figures@plos.org). Please note that Supporting Information files do not need this step.

*In compliance with data protection regulations, you may request that we remove your personal registration details at any time.* [*(Remove my information/details)*](https://gcc02.safelinks.protection.outlook.com/?url=https%3A%2F%2Fwww.editorialmanager.com%2Fpone%2Flogin.asp%3Fa%3Dr&data=04%7C01%7Cjalexand%40usgs.gov%7C58c1bfabeb204b4b0bbf08d93fc3cba3%7C0693b5ba4b184d7b9341f32f400a5494%7C0%7C0%7C637610932448176777%7CUnknown%7CTWFpbGZsb3d8eyJWIjoiMC4wLjAwMDAiLCJQIjoiV2luMzIiLCJBTiI6Ik1haWwiLCJXVCI6Mn0%3D%7C1000&sdata=gBW95Xe0wE3zRQ3WBZlnGyvhRzmQ9ImIZmMM0bqw8Xs%3D&reserved=0)*. Please contact the publication office if you have any questions.*

USGS review and author responses below here

**Alexander et al. Streamflow and spring migration**

**Review by Dr. Tabitha Graves 7/30/21**

Overall this manuscript is well constructed, with appropriate statistical approaches and valid inference. Most suggestions are minor, with a few especially targeted to improve clarity of the stats.

Only major change is to add methods of what is currently called ‘simulation’ to the methods section. I - am unclear what is being simulated however – is this a simulation of snowmelt? My understanding is that if using historic or future records and a model, the correct term is ‘projection’. I think you projected historic migration dates to evaluate how these may have changed over time, but perhaps there was also a simulation component on the hydrology side?

[AUTHOR RESPONSE]: We agree that the term ‘simulation’ is confusing, and not necessary. For context, it is a term that is legacy from a Bayesian stats course I took, and I need to stop using it. I have replaced the term ‘simulation’ with the term ‘prediction’ everywhere in the text. I did not use ‘projection’ ‘prediction’ was already in use in the text, and because it is basically synonymous with ‘projection’.

Would also suggest refer back to sample size in discussion. N=18 years is pretty small, so it is striking that have some significant and useful results given this relatively small sample size.

[AUTHOR RESPONSE]: We agree that referring to the sample size, as well as other limitations of the study is in order. We have added an entire paragraph in the discussion to address this, including a direct reference to sample size.

- [NEW 4^th^ paragraph in Discussion] “*Although our model parameter estimates are significant and accurately reproduce migration behaviors in the NAR and SAR herds, our sample size was relatively small (n=17), and there are assumptions in our models which add complexity to interpretation of our results. The modeled prediction of a median shift in migration initiation of 8 days earlier than the mid-20th century for groups of female mule deer is just beyond the calculated error of the best model (RMSE = 7.4 days). That model relied only on the timing of snowmelt initiation, and thus was strongly influenced by the underlying, but significant, temporal trends of that data. Modeling temporal trends in the timing of female mule deer migration over the past 85 years required assumptions that these herds have been responding to the same environmental cues over that period, that no major shifts in migratory behaviors have occurred (i.e. switch between resident and migratory), and that locations and distances between each herd's summer and winter feeding grounds has not changed. Given that land uses, land and herd management, and vegetation across this landscape has undoubtedly changed over that time period, this assumption is potentially naive. Research by Sawyer et al. (2019)\cite{sawyer2019migratory} has shown that some ungulates, particularly mule deer in the mountainous western United states, have little plasticity in migratory routes, indicating some of our assumptions are reasonable for these species, but may not hold for species with higher migratory route plasticity. Recent modeling by Barnhardt et al. (2020) \cite{barnhart2020} has indicated that snowmelt timing and rate are modulated by plant-available water and available energy, which adds complexity to the assumption that the response of spring plant phenology to snowmelt has remained constant over the modeled historical period. Nonetheless, the extensive and continuous periods of record at some streamgages invites potential for investigating temporal changes in migration initiation that may assist in understanding population dynamics of migrating ungulates which exploit the green wave.*”

This work adds an important tool to the field of migration ecology. Great work!

Minor suggestions follow.

Abstract:

Suggest clarify that you are referring to the ‘timing’ of migration or temporal patterns versus just patterns… which could refer to spatial or temporal patterns.

[AUTHOR RESPONSE]: Agree we have added ‘temporal’ in front of ‘patterns’.

First sentence has linked adjectives- recommend revising, removing ‘so-called’ … maybe something like

.. of NA migrating animals follow the ‘green wave’ of the flush of new vegetation that coincides..

[AUTHOR RESPONSE]: Agree, we have changed the sentence to read “In mountainous and high latitude regions, migratory animals exploit green waves of emerging vegetation coinciding with rising daily mean temperatures initiating snowmelt across the landscape.

Nearly as well in potential to forcast future migrations

[AUTHOR RESPONSE]: The purpose of this comment is unclear.

L22- what is meant by latency here? , I think you mean **e.g.,** MODIS?

[AUTHOR RESPONSE]: Latency is a standard term used in the remote sensing world and refers to the dependability and speed of a data stream to retrieve and process data. Counterintuitively, “low latency” means the system can process lots of data, with great speed, and dependability. The opposite is true for ‘high latency’. Yes, we should use ‘e.g.’ instead of ‘i.e.’ because we are citing an example in the MODIS satellite platform.

L26- I had not heard of this metric- (not sure if change needed, just noting).

[AUTHOR RESPONSE]: The citation for this term immediately follows the introduction of the term in the text.

L35- state not capitalized I think?

[AUTHOR RESPONSE]: True. I have made the word ‘State’ lowercase to read ‘state’.

L65- perhaps title first section ‘Overview’ or simply skip this first paragraph as it seems like you go into all of these later on? Also seems like Study Area could precede this paragraph.

[AUTHOR RESPONSE]: True, we have removed all of the text of this section because it is largely redundant. We did, however, move the sentence “*Logistic regression was used because we were most interested in representing migration initiation as a probability. This interest is rooted in a recognition that the response variable, initiation of spring migration in female mule deer, is informed by data from tens of animals, at most, collectively representing individuals numbering in the thousands*.” to the first paragraph of the ‘Modeling’ section.

L67- add citation to gridded temp data and streamflow data

[AUTHOR RESPONSE]: We removed this statement. The data are cited in the ‘Mean basin temperature and derivatives’ section where they are introduced.

L71- I wonder if a survival (cox model) might have been even more useful? (e.g. Mikle et al.) I think probably not a big difference in outcome… mentioning for future reference…

[AUTHOR RESPONSE]: We agree that a survival model would also work well for the problem because it also expresses probability of migration (given that migration has not yet occurred) through the hazard function. We too are not sure the outcome, though, would be different (would depend on choice of survival distribution function).

L132- suggest put transformation after describe the Cayan et al. method as you do with other variables.

[AUTHOR RESPONSE]: We agree. We have moved the description from below the equation, to the second sentence of the first paragraph in the ‘Timing of snowmelt pulse initiation’. It now reads:

“The method of Cayan et al. (2000; [7]) was used to identify the Julian day of the onset of the annual snowmelt pulse ($D_{{sm}_{j}}$) at each streamgage. The method uses the cumulative departure of daily flow from the mean annual discharge, and thus requires the calendar-year mean annual discharge be known *a priori*. For the purposes of…”

L141- should there be a -1 in reference to the i for one of these? Or maybe Dp?

[AUTHOR RESPONSE]: Great catch! Yes, the first variable was supposed to be Dp_smji_ . The change has been made.

L144- do you mean **by the number of individuals migrating** MI(ji)? Or is this an individual level variable? It’s a bit unclear. Looking further down it seems like this is really ‘at least one individual migrating’?

[AUTHOR RESPONSE]: We agree that this statement was unclear. Yes, ‘individual’ means ‘at least one individual’. We have also changed the term ‘herd’ to ‘group’ to be more consistent with the terminology later in the manuscript. The sentence now reads as follows:

*“Two binary response variables were used in our logistic regression modeling, onset of spring migration of groups of mule deer MH_ji_ within a herd, and onset of migration of at least one individual within a herd MI_ji_”*

L146- Curious- did you calculate these dates or are they reported in something else/ used in another paper that you could cite? NSD method can be a little arbitrary in some landscapes and while I think this is okay, might be nicer to cite the other summary/dataset if it exists.

[AUTHOR RESPONSE]: These data are the same as those in Kaufman et al. (2020), which we cite in the text. We have re-worded the text to make this more clear. It now reads:

*“Migration initiation days were identified using the method of net-squared displacement [29], and full descriptions of the migration data, including initiation dates, can be found in Kaufman et. al (2020; [19])”*

L160- add cite for Kendall-Theil

[AUTHOR RESPONSE]: Done.

L182- am not used to an ‘overview’ of the results before diving in- suggest delete and/or move to within the sections instead. Maybe this is a style used in water journals though?

[AUTHOR RESPONSE]: Yes, this is more common to water journals. I have removed it because the audience is ecologists and you indicate it is not common to that audience.

L186- Simulations are not discussed in Methods--- please add material as to why you are doing them and what kind of simulations you ran.

[AUTHOR RESPONSE]: See first author response above regarding the use of the term ‘simulations’.

L190- here the devil is in the details… am sure you used the Kendall-Theil for some reason, but as I’m not familiar with that particular metric, I’m not sure what it’s doing/ why not significant?

[AUTHOR RESPONSE]: In the same way that traditional (parametric) least-squares regression uses a p-value to assess the ‘significance’ of the parameter, the Kendall-Theil method also calculates a p-value for the slope parameter. So the (p<0.5) is simply that, a p-value. I am not sure how to explain this more clearly, other reviewers from the journal (there were 4 of them) did not seem to have any questions regarding this value.

Fig 5- suggest combine A& B into one figure with different colors representing MI and MH.

[AUTHOR RESPONSE]: Although this might save a bit of space, we believe combining them may confuse the reader, so we would prefer to keep them separate.

Table 2- difference between bold and not bold is slight (are all params left of ‘Herd’ significant?

[AUTHOR RESPONSE]: We agree that the difference between bold and normal fonts would ideally be more clear. However, the options for making tables in the LaTex format (which is used by PLOS ONE to streamline final publication) are limited. Yes, all of the values to the left of ‘Herd’ are significant.

L271/2- Is there more variation in initiation for one or the other herd?

[AUTHOR RESPONSE]: The descriptions of differences in herd, including variation of inititation dates between herds, is contained in the section “Characteristics of spring migration of mule deer in the Atlantic Rim herds”.

L293- too many commas.

[AUTHOR RESPONSE]: Agreed, we have removed the commas.

L330- could also be useful for fence management- e.g. when ok to put the ‘take down’ style of fences back up…

[AUTHOR RESPONSE]: Agreed, inserted the phrase “migration fencing management” in the last paragraph of the Introduction, where other potential uses are also listed. We think it is more appropriate there.
