# Supplementary material for: Testing the potential of streamflow data to predict spring migration of an ungulate herds
Source: PLoS One. 2022 Jan 21;17(1):e0262078. doi: 10.1371/journal.pone.0262078 (PMC8782492; doi:10.1371/journal.pone.0262078)
Supplement: Attachment — Submitted filename: Response to Reviewers.docx [file pone.0262078.s005.docx]

PONE-D-20-40787R2
Testing the potential of streamflow data to predict spring migration of an ungulate
PLOS ONE

Dear Editors,

My co-authors and I are happy to re-submit our manuscript ‘Testing the potential of streamflow data to predict spring migration of an ungulate’ to PLOS ONE. We have accepted all suggestions by the reviewers, include those by reviewer #3 that we had previously rejected.

Please find our responses to reviewers below in red font, each identified with the title ‘[Author response]’. Changes to the manuscript can be found in the document “Revised Manuscript with Track Changes.pdf”, where red strikethrough text indicates removal, and blue text indicates insertion. Because the manuscript was written in Overleaf in the LaTex language, we used the program ‘LaTexDiff’ to track changes. This program is the only option for tracking changes in these types of files, and it sometimes has problems. The contents of table 2, for example, do not appear at all in the track changes document. The only changes to that table were removal of the model characteristics for individual deer (at request of reviewer #3) and associated descriptive elements in the footer. Figure 6 is the only figure that has changed due to peer-review comments. That figure now has a single pane instead of 2 panes, and only shows probability regions for herds of deer.

We hope you find the revised manuscript greatly improved, and suitable for acceptance for publication in PLOS ONE.

Best,

Jason S. Alexander PhD

---------------REVIEW COMMENTS AND RESPONSES BELOW-------------------------------

1. If the authors have adequately addressed your comments raised in a previous round of review and you feel that this manuscript is now acceptable for publication, you may indicate that here to bypass the “Comments to the Author” section, enter your conflict of interest statement in the “Confidential to Editor” section, and submit your "Accept" recommendation.

Reviewer #3: (No Response)

Reviewer #4: All comments have been addressed

2. Is the manuscript technically sound, and do the data support the conclusions?

The manuscript must describe a technically sound piece of scientific research with data that supports the conclusions. Experiments must have been conducted rigorously, with appropriate controls, replication, and sample sizes. The conclusions must be drawn appropriately based on the data presented.

Reviewer #3: Yes

Reviewer #4: Yes

3. Has the statistical analysis been performed appropriately and rigorously?

Reviewer #3: N/A

Reviewer #4: Yes

4. Have the authors made all data underlying the findings in their manuscript fully available?

The [PLOS Data policy](https://gcc02.safelinks.protection.outlook.com/?url=http%3A%2F%2Fwww.plosone.org%2Fstatic%2Fpolicies.action%23sharing&data=04%7C01%7Cjalexand%40usgs.gov%7Caa943fa8280049c99e9f08d9b4e553d0%7C0693b5ba4b184d7b9341f32f400a5494%7C0%7C0%7C637739719326046937%7CUnknown%7CTWFpbGZsb3d8eyJWIjoiMC4wLjAwMDAiLCJQIjoiV2luMzIiLCJBTiI6Ik1haWwiLCJXVCI6Mn0%3D%7C2000&sdata=y3J7oB38j%2BrKFkp0BQpIDulIHgbKUxHNmhp63H%2FGTrs%3D&reserved=0) requires authors to make all data underlying the findings described in their manuscript fully available without restriction, with rare exception (please refer to the Data Availability Statement in the manuscript PDF file). The data should be provided as part of the manuscript or its supporting information, or deposited to a public repository. For example, in addition to summary statistics, the data points behind means, medians and variance measures should be available. If there are restrictions on publicly sharing data—e.g. participant privacy or use of data from a third party—those must be specified.

Reviewer #3: Yes

Reviewer #4: Yes

5. Is the manuscript presented in an intelligible fashion and written in standard English?

PLOS ONE does not copyedit accepted manuscripts, so the language in submitted articles must be clear, correct, and unambiguous. Any typographical or grammatical errors should be corrected at revision, so please note any specific errors here.

Reviewer #3: Yes

Reviewer #4: Yes

6. Review Comments to the Author

Please use the space provided to explain your answers to the questions above. You may also include additional comments for the author, including concerns about dual publication, research ethics, or publication ethics. (Please upload your review as an attachment if it exceeds 20,000 characters)

-------------------------REVIEWER # 3 NEW COMMENTS BELOW-------------------------------

Reviewer #3: Testing the potential of streamflow data to predict spring migration of an ungulate
PONE-D-20-40787R1


The authors made a great job and addressed most issues I presented in the previous review step, substantially enhancing the clarity of the manuscript. I believe that only one point remains problematic, and it may be addressed to further improve the manuscript quality before publication. Moreover, I have a facultative suggestion to highlight a further (minor) result of the manuscript. Below you can find my original comments of the previous reviewing step (only those not fully solved), authors’ response, and my new comment.

- ORIGINAL COMMENT
Introduction and Discussion chapters miss information regarding cues of ungulate migrations. The reported past and future predictions on mule deer migration onset is justified only if environmental cues (temperature, snowmelt, vegetation growth…all variables that authors cleverly summarized within the streamflow) are the only (or main) cue triggering migration onset. Nevertheless, phenology of northern latitudes ungulate species typically (mostly) relies on photoperiodism, which is independent form environmental changes. This is the reason for global change-driven mismatches between migration and optimal foraging conditions provided within the green wave (e.g., Stenseth and Mysterud 2002; Mysterud 2013) and the consequential loss of fitness in migrating ungulates (mentioned by the authors in L46). Notwithstanding, there are evidence of high environmental plasticity of the migration timing, for example in caribou which mostly trigger migration with snowmelt (Laforge et al. 2021). The results of this manuscript potentially provide a similar evidence, but this issue should be introduced and discussed within its broader chronobiological context.


NEW COMMENT
Well done! I consider this point fully addressed from my point of view. I only have a facultative proposal to highlight another biological meaning of your results. I would suggest adding a consideration in discussion, pointing out that your data also provided further evidence of a northern ungulate regulating its phenology with environmental cues. This issue is indeed gaining growing attention by researchers in very recent times, for instance:

Laforge, M. P., Bonar, M., & Vander Wal, E. (2021). Tracking snowmelt to jump the green wave: phenological drivers of migration in a northern ungulate. Ecology, 102(3), e03268.

Brogi, R., Merli, E., Grignolio, S., Chirichella, R., Bottero, E., & Apollonio, M. (2021). It is time to mate: population-level plasticity of wild boar reproductive timing and synchrony in a changing environment. Current Zoology.

[AUTHOR RESPONSE]: Thank you for your input! We agree that additional discussion of the importance of identifying phenological plasticity would enrich the discussion. In response to your suggestion, we have added the following paragraph to the discussion [now the 3^rd^ paragraph of the discussion]:

*“The data in our analysis also demonstrate parity with observations of mule deer as so-called green-wave 'surfers', whereby migration is concurrent with the leading edge of vegetation exposure on the landscape [2, 40]. Animals consistently exhibiting surfing behavior might be expected to delay migration until enough snowmelt has occurred to expose and allow some growth of vegetation along their migratory paths [3]. Indeed, simple linear least-squares regression between our calculated day of spring migration onset of NAR and SAR herds and the day of snowmelt onset produced a linear equation with slope of 0.95 days per day and intercept of 20 days. This relation indicates a consistent delay of nearly three weeks between the onset of snowmelt and onset of migration, with a slight decrease in the lag for later snowmelt onset. With these considerations in mind, our analysis further demonstrates the strong phenological cue that snowmelt provides animals in northern and high-altitude climates. This strong coupling is also further evidence of phenological plasticity to changing environmental cues [4, 12, 41]. Such phenological plasticity has also been demonstrated in species native to temperate climates, and may act as a buffer to inevitable environmental changes [42].”*

This additional discussion required addition of three new references:

- Middleton, A.D., Merkle, J.A., McWhirter, D.E., Cook, J.G., Cook, R.C., White, P.J. and Kauffman, M.J., 2018. Green‐wave surfing increases fat gain in a migratory ungulate. *Oikos*, *127*(7), pp.1060-1068.
- Rickbeil GJ, Merkle JA, Anderson G, Atwood MP, Beckmann JP, Cole EK, Courtemanch AB, Dewey S, Gustine DD, Kauffman MJ, McWhirter DE. Plasticity in elk migration timing is a response to changing environmental conditions. Global change biology. 2019 Jul;25(7):2368-81.
- Brogi R, Merli E, Grignolio S, Chirichella R, Bottero E, Apollonio M. It is time to mate: population-level plasticity of wild boar reproductive timing and synchrony in a changing environment. Current Zoology. 2021 Sep 17.

- ORIGINAL COMMENT
The onset of spring migration by individuals (MI) is biased by the variability in the number of individuals monitored for each year and herd. With the described method, larger samples will inevitably have a higher likelihood to pick smaller minimum values (in this case, earlier dates). MIji is in other words not comparable among years and herds (and indeed seems anticipated in SAR, the herd with more monitored individuals). As individual and herd migration dates are inconsistent (i.e., when comparing SAR and NAR) and predictions have been rightly based only on herd migration (MH) I would suggest removing any mention of MI from the manuscript.

Reviewer #4 NEW COMMENT
I acknowledge authors’ effort to run the bootstrap, but I think that this point is still problematic. The assertion that the smaller sample size is more likely to affect the
precision of the estimate, rather than the accuracy, does not apply here since authors did not calculate the MEAN but the MINIMUM date in which the first individual start migrating. Results of bootstrap are, in my opinion, not fully satisfying. Yes, the average date of SAR seems to remain anticipated in respect to NAR watching bootstrap simulations for over 15 collared animals, but NAR population had typically fewer monitored individuals (min 1, max 16, most years below 11, Tab. 1). Thus, the most informative part of the plot is that below 11 collared individuals, which seems to show a substantial overlap between NAR and SAR. Moreover, a general pattern of decreasing date of migration for increasing number of collared individuals (either real or bootstrapped) seems to emerge for SAR in the provided plot.
Finally, the differences between SAR and NAR migration dates are substantial only for MI and almost negligible for MH (Fig 5 A vs B). How do authors explain this inconsistency among the two measures? Since predictions have been rightly based only on herd migration (MH), my suggestion remains to remove any mention of MI from the manuscript.

[AUTHOR RESPONSE]: Although we disagree with the reviewer’s assertion that the weak bias in the models of individual mule deer would warrant removal of those models and discussions, we recognize that the reviewer’s concerns would also be concerns of some portion of PLOS ONE’s audience. Thus, we have removed the models of individual deer migration. The biggest change was in table 2, which presented the model parameter estimates. We did, however, keep the presentation of range of observed migration initiation dates in the “Characteristics of spring migration of mule deer in the Atlantic Rim herds” section and Table 1 (which lists the data characteristics) because we thought this discussion was relevant evidence that our method of using ‘groups’ as representative of herd behavior was valid. Thus, figure 5, which shows minimum migration dates of individuals and groups in each herd, has also not changed because it demonstrates that, although there is not parity between individuals, there is for groups. Figure 6, which showed the 95% credible ranges of probability of migrations from our models now has only one pane, which only shows the model predictive results for the herds.

-------------------------REVIEWER # 4 NEW COMMENTS BELOW-------------------------------

Reviewer #4: I am happy with the response of the authors to my comments. Reading the ms again, I only have three very minor comments:
L31 ‘Since ‘host’ is typically used in the field of disease ecology to denote the host for pathogens, I would attain to a more intuitive expression, e.g. ‘…found that several/many ungulate…”

[AUTHOR RESPONSE]: Agreed. We have changed it to read “*examined surfing behaviors of several species of ungulates*”

L193: 150 or 151?

[AUTHOR RESPONSE]: 151 because 0 to 150 is 151 numbers (because zero is included).
L416: thaytrecord? I guess is a typo...

[AUTHOR RESPONSE]: Yes, typo, good catch. We changed it to ‘that record’.

7. PLOS authors have the option to publish the peer review history of their article ([what does this mean?](https://gcc02.safelinks.protection.outlook.com/?url=https%3A%2F%2Fjournals.plos.org%2Fplosone%2Fs%2Feditorial-and-peer-review-process%23loc-peer-review-history&data=04%7C01%7Cjalexand%40usgs.gov%7Caa943fa8280049c99e9f08d9b4e553d0%7C0693b5ba4b184d7b9341f32f400a5494%7C0%7C0%7C637739719326046937%7CUnknown%7CTWFpbGZsb3d8eyJWIjoiMC4wLjAwMDAiLCJQIjoiV2luMzIiLCJBTiI6Ik1haWwiLCJXVCI6Mn0%3D%7C2000&sdata=H%2FlGfQM4YkQvJmvYRKupfTG7xUKWXCchT4BBPb5h0pQ%3D&reserved=0)). If published, this will include your full peer review and any attached files.


If you choose “no”, your identity will remain anonymous but your review may still be made public.


**Do you want your identity to be public for this peer review?** For information about this choice, including consent withdrawal, please see our [Privacy Policy](https://gcc02.safelinks.protection.outlook.com/?url=https%3A%2F%2Fwww.plos.org%2Fprivacy-policy&data=04%7C01%7Cjalexand%40usgs.gov%7Caa943fa8280049c99e9f08d9b4e553d0%7C0693b5ba4b184d7b9341f32f400a5494%7C0%7C0%7C637739719326056894%7CUnknown%7CTWFpbGZsb3d8eyJWIjoiMC4wLjAwMDAiLCJQIjoiV2luMzIiLCJBTiI6Ik1haWwiLCJXVCI6Mn0%3D%7C2000&sdata=ZyaOxG1Q2sV7gpPT6%2BxwexpYW83WEWUV7ARMPGmHxd4%3D&reserved=0).

Reviewer #3: **Yes:** Rudy Brogi

Reviewer #4: No
